# SPRR3 Contributes to Aggressiveness of Pancreatic Cancer Cells via NF-*κ*B Signaling Pathway

**DOI:** 10.1155/2023/7518744

**Published:** 2023-01-13

**Authors:** Yuankang Xie, Heping Li, Baiyin Zhong, Xiansen Zhu, Rong Ye, Binhui Xie, Jianhong Zhang

**Affiliations:** ^1^Department of Hepatobiliary Surgery, The First Affiliated Hospital of Gannan Medical University, Ganzhou 341000, China; ^2^Ganzhou Key Laboratory of Hepatocellular carcinoma, The First Affiliated Hospital of Gannan Medical University, Ganzhou 341000, China; ^3^Department of Medical Oncology, The First Affiliated Hospital of Sun Yat-sen University, Guangzhou 510080, China; ^4^Department of Pathology, The First Affiliated Hospital of Gannan Medical University, Ganzhou 341000, China; ^5^Department of General Surgery III, The First Affiliated Hospital of Gannan Medical University, Ganzhou 341000, China

## Abstract

Pancreatic cancer remains a deadly solid tumor with worst survival, and a better understanding of the mechanisms of carcinogenesis of pancreatic cancer is critical to promote the survival of patients with pancreatic cancer. qPCR and western blot assay were used to determine the expression of SPRR3 in pancreatic cancer. Anchorage-independent growth ability, BrdU labeling, Transwell assay, and in vivo experiment were used to examine the functions of SPRR3 in aggressiveness of pancreatic cancer. Luciferase reporter assay, nucleoplasmic-separation technique, qPCR, and western blot assay were used to investigate the mechanism of SPRR3 regulating aggressiveness of pancreatic cancer. Our results showed that SPRR3 was significantly increased in pancreatic cancer, which resulted in poor survival for patients with pancreatic cancer. Further analysis showed that overexpression of SPRR3 contributed to anchorage-independent growth ability, growth rate, and invasion ability of pancreatic cancer cells. While, knockdown of SPRR3 showed the reverse results. Mechanistically, overexpression of SPRR3 can promote the transcription of NF-*κ*B pathway, nuclear accumulation of p65, and mRNA levels of NF-*κ*B pathway downstream genes. But, knockdown of SPRR3 induced the reverse results. The above findings clarified the important roles of SPRR3 in the progression of pancreatic cancer through NF-*κ*B pathway. And targeting SPRR3 might be an effective strategy to therapy pancreatic cancer.

## 1. Introduction

Despite recent advances in detection, surgical techniques, and therapy, pancreatic cancer remains a deadly solid tumor with worst survival [1]. Five-year survival rate of pancreatic cancer only increased from 3% to 8% over the past 40 years [2]. The reasons for such dismal survival were lack of specific symptoms, early detection, and effective therapy strategy [3]. Therefore, it is essential to investigate the mechanisms of pancreatic cancer tumorigenesis and progression and find out the effective therapy strategy.

NF-*κ*B pathway plays important functions in multiple physiological and pathological processes. According to activating mechanisms, there are two types of NF-*κ*B pathways, the canonical and noncanonical pathway [4, 5]. The crucial step of the canonical NF-*κ*B pathway is phosphorylation-dependent activation of the IKKs (I*κ*B kinases) complex [6, 7]. In the steady state, transcript factor NF-*κ*B is sequestered in the cytoplasm by the I*κ*B. In the agitated state, the activated IKKs phosphorylate the inhibitory I*κ*B protein, which is subsequently ubiquitination-dependently degraded through proteasome. The degradation of I*κ*B lets NF-*κ*B liberate, consequently translocate to nucleus and activate the target genes [8]. Increasing evidences showed that NF-*κ*B pathway is closely correlated with the incidence of pancreatic cancer. For example, Wang et al. showed that overexpression of GPR87 contributes to pancreatic cancer aggressiveness through NF-*κ*B pathway ([9]). Ren et al. showed that lncRNA PLACT1 can foster the progression of pancreatic cancer through sustained activation of NF-*κ*B pathway ([10]). Yu et al. showed that oncogenic TRIM31 promotes gemcitabine resistance of pancreatic cancer through activating NF-*κ*B pathway [11]. Therefore, a better understanding of NF-*κ*B signaling pathway in pancreatic cancer might provide effective therapeutic strategy for patients with pancreatic cancer.

SPRR3, a member of the large family of the small proline-rich proteins (SPRRs), is located in the epidermal differentiation complex on chromosome 1q21 [12]. Abnormal expression of SPRR3 has been reported to be identified in multiple cancers. For example, Li et al. showed that dysregulation of SPRR3/miR-876-3p signaling axis is available to carcinogenesis of non-small-cell lung cancer ([13]). Cho et al. showed that increased expression of SPRR3 promotes colorectal tumorignesis ([14]). Kim et al. showed that SPRR3 contributes to the proliferation of breast cancer cell via promoting p53 degradation [15]. However, the expression, function, and mechanism of SPRR3 in pancreatic cancer remain unclear.

Herein, we aim to investigate the expression, function, and mechanism of SPRR3 in pancreatic cancer and make an attempt to clarify the underlying mechanism of carcinogenesis of pancreatic cancer and find the effective therapeutic manner to therapy it.

## 2. Material and Methods

### 2.1. Cell Culture

All cell lines were purchased from Chinese Type Culture Collection, Chinese Academy of Sciences. All the cell lines were cultured using DMEM adding 10% fetal bovine (FBS; HyClone, USA), 100 U/ml penicillin, and 100 *μ*g/ml streptomycin (Sigma-Aldrich, USA).

### 2.2. Establishment of Stable Cell Lines

The plasmids of SPRR3 upregulation, SSR2 downregulation, and corresponding control were designed and synthesized by Guangzhou RiboBio Co., Ltd. (Guangzhou, China). The process of screening stable cell lines was performed according to previously published methods [16].

### 2.3. qRT-PCR Analysis

The total RNA was extracted using TRIzol (Invitrogen, USA). And total cDNA was synthesized using Transcriptor First Strand cDNA Synthesis Kit (Roche, Germany) according to the manufacturer's directions. qPCR was performed on a 7500 fast real time PCR system (Applied Biosystems, USA) using the SYBR Green PCR Kit (Invitrogen, USA). The primers in the present study are shown in Table 1.

### 2.4. Western Blot Assay

Total protein was obtained by radioimmunoprecipitation assay (RIPA) lysis buffer (Beyotime Biotechnology, China). Western blot was performed using the extracted proteins according to previously described methods [17]. Briefly, protein was separated using 10.5% polyacrylamide gels and subsequently transferred onto PVDF membranes. Then, the membranes were probed with primary antibodies and incubated with horseradish peroxidase- (HRP-) conjugated secondary antibody. *α*-Tubulin was used as the loading control of total protein.

### 2.5. Anchorage-Independent Growth Assay

Anchorage-independent growth assay was performed following the methods described previously [18]. Firstly, complete medium containing 1% agar was added into the 6-well cell plates. Subsequently, 0.5 × 10^3^ cells that were suspended using complete medium containing 0.3% low melt agarose were added into the top of cell plates. 10 days later, the colonies with diameter larger than 100 *μ*m were counted.

### 2.6. Bromodeoxyuridine (BrdU) Labeling

Cells were seeded onto coverslips, which were put into 24-well plates. 24 h later, the cells were incubated using BrdU for 1 h and subsequently probed using anti-BrdU antibody for 2 h (Upstate Biotechnology, USA). Images of cells were collected under a laser scanning microscope (Carl Zeiss, Germany).

### 2.7. Transwell Matrix Penetration Assay

The Transwell filter chamber was coated using Matrigel (BD Biosciences, USA). The coated chamber was subsequently put into 24-well plate that was added cell medium. Then, 1 × 10^4^ cells were suspended using medium containing 10% FBS (HyClone, USA) and seeded into the coated chamber. And then, the cell plates were maintained in a humidified cell incubator for 24 h. The cells inside the upper chamber were removed using cotton swabs. The remaining cells were fixed using 1% paraformaldehyde for 10 min and stained using hematoxylin for 5 min. Finally, the invaded cells were counted using microscope (CKX41; Olympus) in 10 randomly chosen fields.

### 2.8. Xenografted Tumor Model

In the subcutaneous tumor model, BALB/c nude mice were randomly divided into two groups. Every mice were inoculated subcutaneously with 2 × 10^6^ PANC-1/shRNA-V or PANC-1/SPRR3 sh#2 cells, respectively, in the dorsal flank per mouse. Tumor was monitored by measurements of the length and width, and the tumor volume was calculated following the equation (*L* × *W*^2^)/2. 30 days later, all mice were euthanized and dissected. Tumors were excised and weighed. All experimental procedures were approved by the Institutional Animal Care and Use Committee of the First Affiliated Hospital of Gannan Medical University.

### 2.9. Statistical Analysis

Statistical analyses were performed using the SPSS version 19.0 statistical software package. The data are present as the mean ± standard deviation. Student's paired *t*-test was used to analyze the statistical difference between paired tissues, and comparisons among more than two groups were analyzed using variance (ANOVA) followed by Dunnett's test. *P* < 0.05 was considered as statistically significance.

## 3. Results

### 3.1. High Levels of SPRR3 Predicted Poor Survival for Patients with Pancreatic Cancer

**Figure 1:**
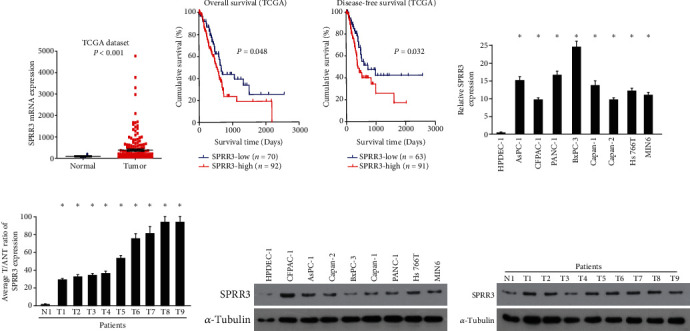
High levels of SPRR3 predicted poor survival for patients with pancreatic cancer. (a) Abnormally high expression of SPRR3 was discovered using The Cancer Genome Atlas (TCGA) dataset. (b) High levels of SPRR3 predicted poor overall survival for patients with pancreatic cancer. (c) High levels of SPRR3 predicted poor disease-free survival for patients with pancreatic cancer. (d) The mRNA levels of SPRR3 in indicated pancreatic cancer cells. (e) The mRNA levels of SPRR3 in pancreatic cancer tissues. (f) The protein levels of SPRR3 in indicated pancreatic cancer cells. (g) The protein levels of SPRR3 in pancreatic cancer tissues. ^∗^*P* < 0.05.

Abnormally high expression of SPRR3 was discovered using The Cancer Genome Atlas (TCGA) dataset (Figure 1(a)). The correlation of SPRR3 levels and survival was further confirmed. The analysis showed high levels of SPRR3 predicted poor overall survival (Figure 1(b)) and poor disease-free survival (Figure 1(c)) for patients with pancreatic cancer. Consistently, the mRNA (Figure 1(d)) and protein expression (Figure 1(f)) of SPRR3 were markedly higher in pancreatic cancer cell lines AsPC-1, CFPAC-1, PANC-1, BxPC-3, Capan-1, Capan-2, Hs 766 T, and MIN6 than in normal human pancreatic ductal epithelial cells (HPDECs). Moreover, the same situation was illustrated in fresh tissues. qPCR (Figure 1(e)) and western blot (Figure 1(g)) assay showed SPRR3 levels were significantly increased in pancreatic cancer tissues compared with normal pancreatic tissues. These results confirmed the analysis using TCGA dataset, suggesting that SPRR3 was upregulated in pancreatic cancer.

### 3.2. Overexpression of SPRR3 Contributed to the Aggressiveness of Pancreatic Cancer

**Figure 2:**
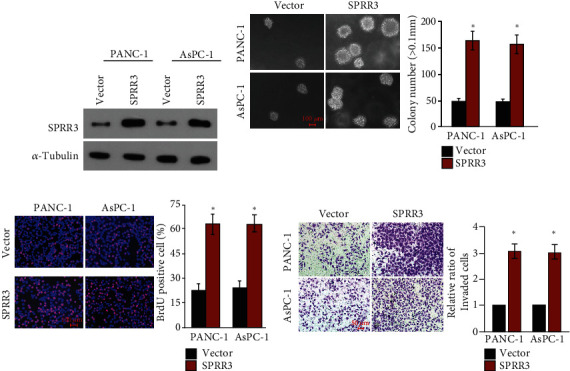
Overexpression of SPRR3 contributed to the aggressiveness of pancreatic cancer cells. (a) The SPRR3 protein levels in stable cell lines overexpressing SPRR3. (b) The representative images and quantitative analysis of anchorage-independent growth assay in stable cell lines overexpressing SPRR3. (c) The representative images and quantitative analysis of BrdU labeling in stable cell lines overexpressing SPRR3. (d) The representative images and quantitative analysis of Transwell invasion assay in stable cell lines overexpressing SPRR3. ^∗^*P* < 0.05.

To investigate the function of SPRR3 upregulation in pancreatic cancer, PANC-1 and AsPC-1 cell lines with stable overexpression of SPRR3 were established (Figure 2(a)). The anchorage-independent growth assay showed that colonies formed by SPRR3-overexpessing cells were more and larger than that formed by control cells (Figure 2(b)). Moreover, BrdU incorporation assay showed that overexpression of SPRR3 significantly facilitated the growth rate of pancreatic cancer cells (Figure 2(c)). Furthermore, Transwell assay showed that overexpression of SPRR3 promoted the invasion ability of pancreatic cancer cells (Figure 2(d)).

Altogether, our analysis suggested that upregulation of SPRR3 facilitated the aggressiveness of pancreatic cancer cells.

### 3.3. Knockdown of SPRR3 Inhibited the Aggressiveness of Pancreatic Cancer Cells

**Figure 3:**
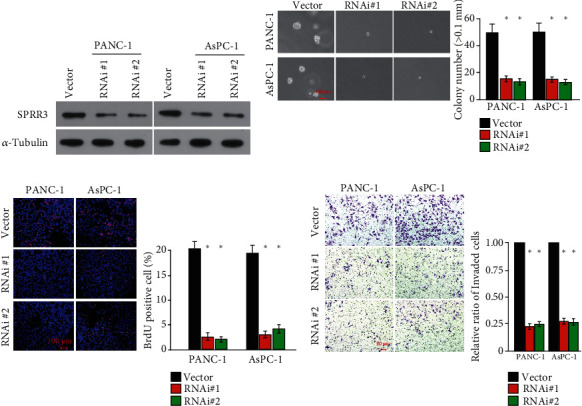
Knockdown of SPRR3 inhibited the aggressiveness of pancreatic cancer cells. (a) The SPRR3 protein levels in stable cell lines with SPRR3 knockdown. (b) The representative images and quantitative analysis of anchorage-independent growth assay in stable cell lines downregulating SPRR3. (c) The representative images and quantitative analysis of BrdU labeling in stable cell lines downregulating SPRR3. (d) The representative images and quantitative analysis of Transwell invasion assay in stable cell lines downregulating SPRR3. ^∗^*P* < 0.05.

**Figure 4:**
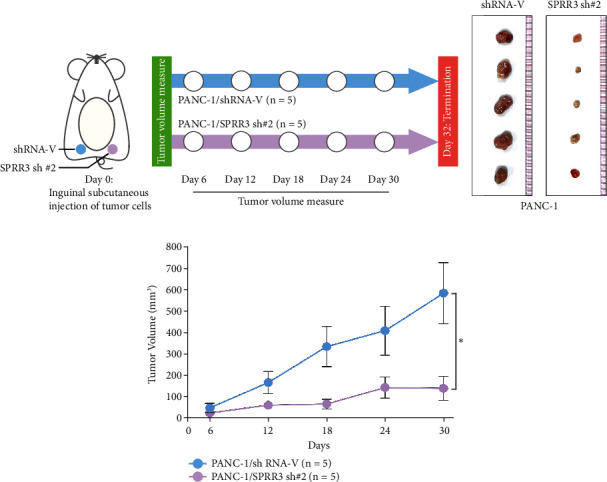
In vivo experiment showed that knockdown of SPRR3 inhibited the tumor formation of pancreatic cancer cells. (a) Schematic diagram of in vivo experiment. (b) The mice tumor formed by PANC-1 cells. (c) Tumor volume of mice tumor. ^∗^*P* < 0.05.

We also established the stable cell lines with SPRR3 knockdown to explore the function of SPRR3 in the progression of pancreatic cancer (Figure 3(a)). Our results showed that anchorage-independent growth ability of SPRR3 downregulating pancreatic cancer cells significantly reduced compared with control cells (Figure 3(b)). Moreover, BrdU incorporation assay showed that knockdown of SPRR3 dramatically inhibited the growth rate of pancreatic cancer cells (Figure 3(c)). In addition, the invasion ability of pancreatic cancer was significantly reduced in SPRR3-silenced pancreatic cancer cells (Figure 3(d)). We performed the in vivo experiments (Figures 4(a)–4(c)). The in vivo experiments showed that the tumors formed by SPRR3-knockdown cells are smaller than that formed by control cells. Taken together, our results showed that knockdown of SPRR3 inhibited the aggressiveness of pancreatic cancer cells.

### 3.4. SPRR3 Regulated NF-*κ*B Signaling in Pancreatic Cancer

**Figure 5:**
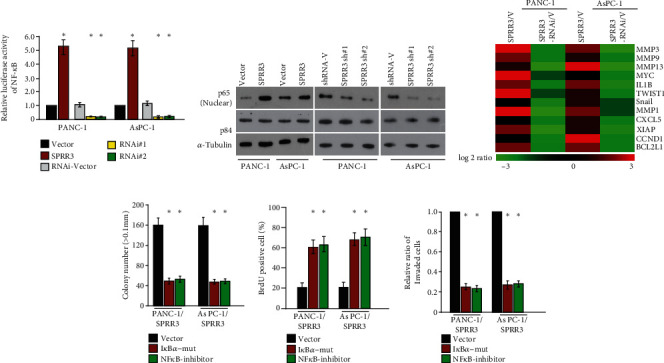
SPRR3 regulated the NF-*κ*B signaling in pancreatic cancer. (a) NF-*κ*B luciferase reporter assay showed that NF-*κ*B transcription activity was dramatically elevated in SPRR3-upregulating cells, while reduced in SPRR3-silenced cells. (b) The levels of nuclear p65 in indicated cell lines. (c) The mRNA levels of NF-*κ*B downstream genes. (d) NF-*κ*B inhibitor inhibited colonies formed in soft agar of SPRR3-overexpressing cells. (e) NF-*κ*B inhibitor inhibited BrdU positive cells of SPRR3-overexpressing cells. (f) NF-*κ*B inhibitor inhibited invasive ability of SPRR3-overexpressing cells. ^∗^*P* < 0.05.

Since NF-*κ*B signaling plays an important role in aggressiveness of pancreatic cancer, we subsequently investigate whether SPRR3 regulated the NF-*κ*B signaling in pancreatic cancer. NF-*κ*B luciferase reporter assay showed that NF-*κ*B transcription activity was dramatically elevated in SPRR3-upregulating cells while reduced in SPRR3-silenced cells (Figure 5(a)). Besides, the levels of nuclear p65 were markedly increased in SPRR3-upregulating cells but reduced in SPRR3-silenced cells (Figure 5(b)). In addition, the levels of NF-*κ*B downstream genes were significantly increased in SPRR3-upregulating cells but decreased in SPRR3-knockdown cells (Figure 5(c)). These results inferred that SPRR3 enhanced the activation of NF-*κ*B pathway.

Finally, the blockage of NF-*κ*B pathway using NF-*κ*B inhibitor inhibited colonies formed in soft agar (Figure 5(d)), BrdU positive cells (Figure 5(e)), and invaded cells (Figure 5(f)) of SPRR3-overexpressing cells, suggesting that activation of NF-*κ*B is essential for SPRR3 regulating aggressiveness of pancreatic cancer cells.

### 3.5. Clinical Correlation of SPRR3 Levels and NF-*κ*B Activation in Pancreatic Cancer

**Figure 6:**
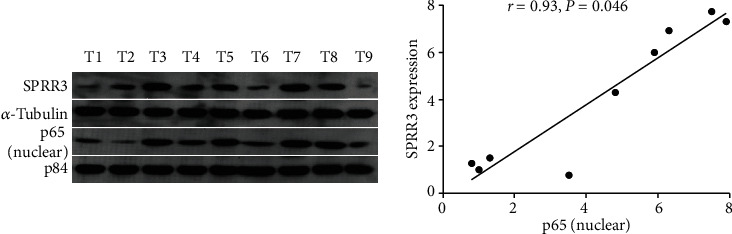
Clinical correlation of SPRR3 levels and NF-*κ*B activation in pancreatic cancer. The representative bands (left panel) and correlation analysis (right panel) of SPRR3 and p65.

Besides, we determined whether SPRR3 contributed to p65 accumulation in clinical samples. As shown in Figure 6, SPRR3 expression was positively related with nuclear p65 levels (*r* = 0.93; *P* = 0.046), which further supported that SPRR3 facilitated the aggressiveness of pancreatic cancer cells and the activation of NF-*κ*B signaling.

## 4. Discussion

Our study provided evidence for a new link of SPRR3 and NF-*κ*B pathway in pancreatic cancer. Firstly, our analysis showed that SPRR3 was significantly increased in pancreatic cancer and closely related with poor survival for patients with pancreatic cancer. Secondly, cell function tests showed that SPRR3 elevated anchorage-independent growth ability, BrdU positive cells, and invasion ability of pancreatic cancer cells. While, downregulation of SPRR3 showed the reverse results. Finally, molecular mechanism analysis suggested SPRR3 induced the activation of NF-*κ*B pathway. The above findings clarified the important roles of SPRR3 in progression of pancreatic cancer through NF-*κ*B pathway.

Pancreatic cancer is still one of the deadliest cancers. Despite much improvement in the survival rates for patients with other cancer types, the survival rates of patients with pancreatic cancer have almost unchanged since 1960s [19]. Patients with pancreatic cancer often diagnosed at advanced stage, and most therapy manners are ineffective, which result in poor survival for patients with pancreatic cancer [20]. Therefore, understanding the mechanisms of carcinogenesis of pancreatic cancer is essential. Our results showed that SPRR3 was significantly overexpressed in pancreatic cancer, which contributed to aggressiveness of pancreatic cancer. Corresponding, knockdown of SPRR3 inhibited the aggressiveness of pancreatic cancer. Our results extended the mechanisms of pancreatic cancer carcinogenesis and provided evidence that targeting SPRR3 might be an effective strategy to therapy pancreatic cancer.

It has been reported that inflammation takes a central role in pancreatic cancer development, and NF-*κ*B pathway is characterized as a key pathway of inflammation, and frequently dysregulated in pancreatic cancer [21–23]. Our present study showed that SPRR3 can elevate the activity of NF-*κ*B pathway. The activation of NF-*κ*B pathway further promotes the transcription of downstream genes that are involved in proliferation, antiapoptosis, metastasis, and so on, which finally contributes to the aggressiveness of pancreatic cancer [24]. Therefore, therapeutic targeting NF-*κ*B pathway has been aggressively pursued for the treatment of a wide range of malignant pathologies in pancreatic cancer [25–27]. Our study showed that NF-*κ*B pathway inhibitor can significantly inhibit the aggressiveness of pancreatic cancer.

Besides, our study leaves much to be desired. For example, the function of SPRR3 in vivo in pancreatic cancer needs to be clarified, and how SPRR3 regulate NF-*κ*B signaling is unclear, and so on. We will continue to clarify these issues in our future study.

## Figures and Tables

**Table 1:** Primers for qPCR.

Primers for qPCR	
SPRR3 real time primer up	TGAACCAGGCAGCATCAAGGTC
SPRR3 real time primer dn	GAAGGACATGGCTCTGGTAGCT
MMP3 real time primer up	CACTCACAGACCTGACTCGGTT
MMP3 real time primer dn	AAGCAGGATCACAGTTGGCTGG
MMP9 real time primer up	GCCACTACTGTGCCTTTGAGTC
MMP9 real time primer dn	CCCTCAGAGAATCGCCAGTACT
MMP13 real time primer up	CCTTGATGCCATTACCAGTCTCC
MMP13 real time primer dn	AAACAGCTCCGCATCAACCTGC
MYC real time primer up	CCTGGTGCTCCATGAGGAGAC
MYC real time primer dn	CAGACTCTGACCTTTTGCCAGG
IL1B real time primer up	CCACAGACCTTCCAGGAGAATG
IL1B real time primer dn	GTGCAGTTCAGTGATCGTACAGG
TWIST1 real time primer up	GCCAGGTACATCGACTTCCTCT
TWIST1 real time primer dn	TCCATCCTCCAGACCGAGAAGG
Snail real time primer up	TGCCCTCAAGATGCACATCCGA
Snail real time primer dn	GGGACAGGAGAAGGGCTTCTC
MMP1 real time primer up	ATGAAGCAGCCCAGATGTGGAG
MMP1 real time primer dn	TGGTCCACATCTGCTCTTGGCA
CXCL5 real time primer up	CAGACCACGCAAGGAGTTCATC
CXCL5 real time primer dn	TTCCTTCCCGTTCTTCAGGGAG
XIAP real time primer up	TGGCAGATTATGAAGCACGGATC
XIAP real time primer dn	AGTTAGCCCTCCTCCACAGTGA
CCND1 real time primer up	TCTACACCGACAACTCCATCCG
CCND1 real time primer dn	TCTGGCATTTTGGAGAGGAAGTG
BCL2L1 real time primer up	GCCACTTACCTGAATGACCACC
BCL2L1 real time primer dn	AACCAGCGGTTGAAGCGTTCCT
GAPDH real time primer up	GGAGCGAGATCCCTCCAAAAT
GAPDH real time primer dn	GGCTGTTGTCATACTTCTCATGG

## Data Availability

The data used to support the findings of this study are available from the corresponding authors upon request.
